# Successful upadacitinib treatment in anti-TNF-refractory intestinal Behçet’s disease: a case report and literature review

**DOI:** 10.3389/fmed.2026.1783949

**Published:** 2026-02-19

**Authors:** Yanfen Shi, Minggang Zhang, Xiaodi Wang, Fang Liu, Jianan Chen, Wenjuan Guo

**Affiliations:** 1 Department of Pathology, China-Japan Friendship Hospital, Beijing, China; 2 Department of Gastroenterology, China-Japan Friendship Hospital, Beijing, China; 3 Department of Clinical Sciences, H. Lee Moffitt Cancer Center & Research Institute, Tampa, FL, United States

**Keywords:** anti-TNF resistance, Behçet’s disease, case report, JAK inhibitors, upadacitinib

## Abstract

**Background:**

Behçet’s disease (BD) is a persistent inflammatory vasculitis affecting various vessel types across multiple organ systems. It clinically presents recurrent oral and genital ulcers, ocular inflammation, and various skin manifestations. Etiology remains multifactorial, involving genetic susceptibility, immune system dysregulation, and environmental triggers such as infections. Intestinal involvement represents a rare but particularly severe form of BD, whose clinical features frequently resemble those of inflammatory bowel diseases (IBD), complicating differential diagnosis and management.

**Case presentation:**

We report a case of a 23-year-old male with progressive postprandial abdominal pain, diarrhea, and marked weight loss. His clinical history was notable for recurrent oral aphthae, genital ulcers, and perianal infections. Colonoscopic examination revealed circumferential ulceration, mucosal edema, contact bleeding, and significant narrowing of the ileocecal lumen. Histopathological analysis indicated chronic active inflammation in the absence of granulomas or definitive vasculitis. Infectious and neoplastic causes were systematically excluded. The patient was diagnosed with intestinal BD. Although initial therapy with infliximab yielded partial clinical improvement, drug-level monitoring revealed suboptimal trough levels, indicating secondary loss of response. Subsequently, treatment was transitioned to the Janus kinase (JAK) inhibitor upadacitinib, which led to full symptom resolution and mucosal healing on follow-up endoscopy.

**Conclusion:**

This case underscores the diagnostic and therapeutic challenges associated with intestinal BD, especially in distinguishing it from Crohn’s disease and addressing resistance to anti-TNF agents. Our findings suggest that JAK inhibitors like upadacitinib may offer a promising alternative for patients with refractory intestinal BD. Incorporating therapeutic drug monitoring into clinical practice allows for personalized, adaptive treatment adjustments, potentially improving long-term outcomes in complex cases.

## Introduction

Behçet’s disease (BD) is a chronic, relapsing, multisystem vasculitis that can involve both arteries and veins of any caliber (1). It affects multiple organ systems including the mucosa, skin, eyes, joints, central nervous system, and gastrointestinal (GI) tract (2). The clinical manifestations of BD vary across different populations and geographical regions. Gastrointestinal involvement, referred to as intestinal BD, is more commonly seen in East Asia and may present with deep mucosal ulcers, strictures, massive hemorrhage, or even intestinal perforation. The most commonly involved site is the ileocecal region.

The pathogenesis of BD is multifactorial and involves a complex interplay of genetic predisposition—particularly HLA-B*51—and environmental triggers such as infectious agents (3). Both innate and adaptive immune responses contribute to disease activity, including hyperactivation of neutrophils and skewing toward Th1 and Th17 responses. Intestinal BD often presents with features that overlap with Crohn’s disease, making diagnosis and management particularly challenging (4).

Conventional therapies for intestinal BD include 5-aminosalicylic acid (5-ASA), corticosteroids, and immunomodulators such as azathioprine or thalidomide. Anti-TNF-*α* agents like infliximab have demonstrated efficacy in inducing and maintaining remission, and are recommended by international guidelines as first-line biologics for refractory cases (5). Managing intestinal BD in patients who fail to respond—either initially or over time—to anti-TNF therapy remains an ongoing clinical challenge (6). This case report summarizes current therapeutic approaches in this context. Janus kinase (JAK) inhibitors such as upadacitinib have emerged as promising therapeutic options through inhibition of inflammatory cytokines including IL-2, IL-6, IFN-*γ*, and IL-17 via the JAK–STAT pathway (7).

Recent case reports have described successful upadacitinib use in anti-TNF–refractory intestinal Behçet’s disease. We report an additional case characterized by stricturing ileocecal disease and infliximab failure despite therapeutic drug monitoring–guided dose intensification, followed by rapid clinical and endoscopic remission with upadacitinib. This case underscores the potential value of JAK inhibitors as alternative therapies in biologic-refractory intestinal BD and highlights the importance of individualized, dynamic treatment strategies guided by clinical response and drug monitoring.

## Case presentation

A 23-year-old male presented with a 5-year history of abdominal pain, which had worsened over the past year. Five years prior to presentation, he experienced persistent dull periumbilical pain after meals, accompanied by increased bowel movements (1–3 times per day) and relief after defecation, occurring one to two times per month. Over the year preceding presentation, the symptoms progressed to daily occurrences, with prolonged episodes of abdominal pain and intermittent diarrhea.

He had a history of recurrent oral ulcers, genital ulcers, and perianal abscesses. During the disease course, he denied fever, alopecia, dry mouth or eyes, arthralgia, Raynaud’s phenomenon, otitis media, sinusitis, or ocular involvement. He experienced a weight loss of approximately 10 kg over the past year. Physical examination revealed scattered acneiform lesions on the face and back, mild tenderness in the right upper abdomen without rebound pain, and no edema in the lower extremities. His body mass index (BMI) was 16.9 kg/m^2^.

Laboratory investigations showed leukocytosis (WBC 14.4 × 10^9^/L), neutrophilia (11.51 × 10^9^/L), thrombocytosis (PLT 380 × 10^9^/L), mild anemia (HGB 106 g/L), elevated high-sensitivity C-reactive protein (hs-CRP 21.3 mg/L), and an ESR of 17 mm/h. Stool occult blood was weakly positive. Autoimmune and vasculitis antibody panels were negative. T-SPOT. TB was negative. CMV-DNA, EBV-DNA, *Clostridium difficile* culture, and tumor markers were all negative.

**Figure 1:**
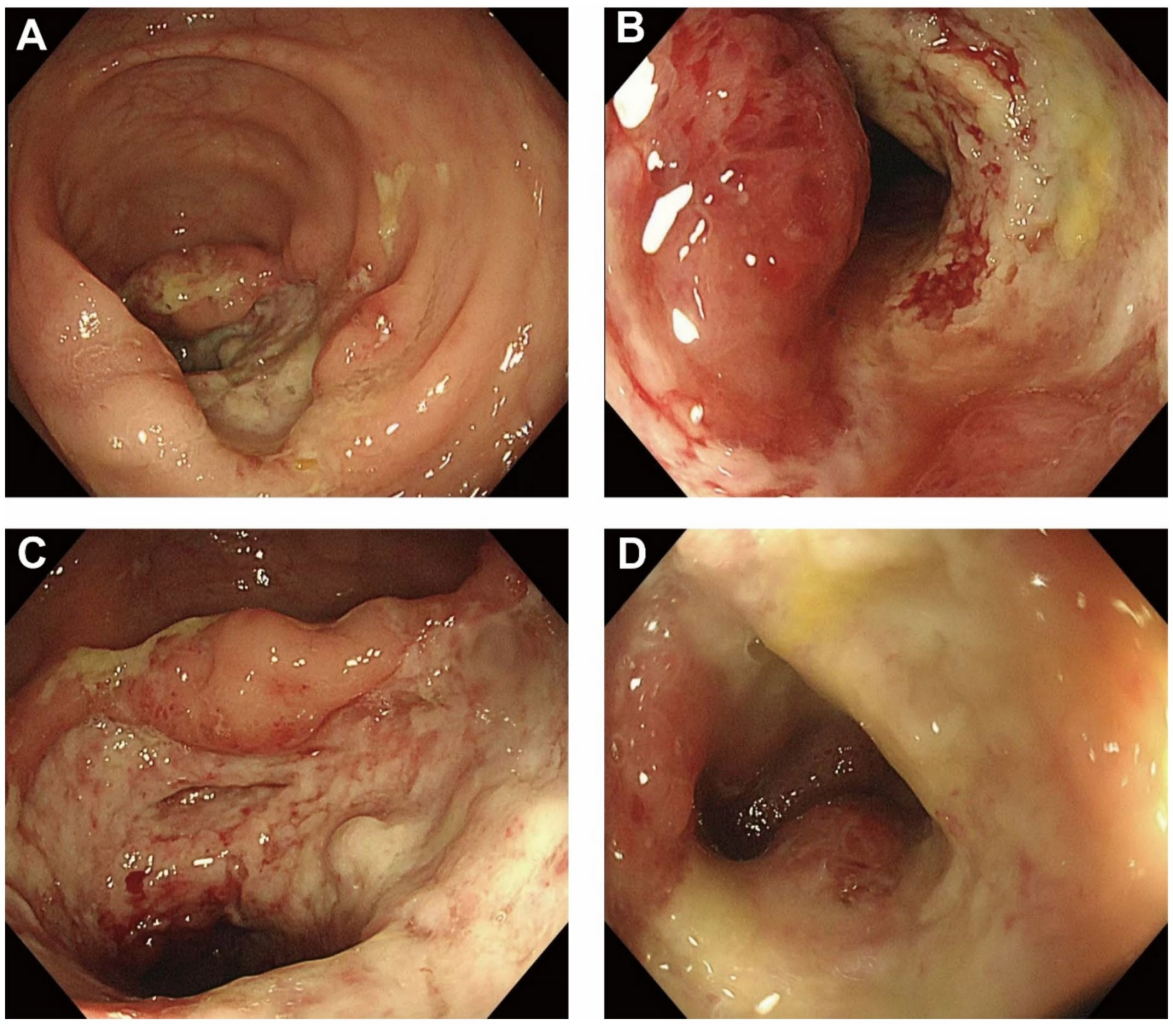
Colonoscopic findings of the ileocecal region at initial diagnosis. **(A)** A deep, circumferential ulcer is observed in the ileocecal area, covered with yellowish-white exudate and surrounded by markedly edematous mucosa. **(B)** Significant luminal narrowing is present at the ulcer site, with increased mucosal friability, preventing further advancement of the endoscope. **(C)** Diffuse mucosal erosion with spontaneous bleeding and a granular appearance is seen; ulcer borders appear irregular. **(D)** A large, severe ulcer with central necrosis, bridging mucosal folds, and raised margins suggests active transmural inflammation.

An esophagogastroduodenoscopy (EGD) revealed chronic non-atrophic gastritis, with mild chronic inflammation and lymphocytic infiltration of the antral mucosa. Colonoscopy identified a circumferential ulcer approximately 70 cm from the anal verge, with clearly defined margins, edematous raised mucosa, thick white exudate on the surface, and contact bleeding. The intestinal lumen was progressively narrowed to about 0.6 cm, preventing further scope advancement (Figure 1). Histopathology revealed acute and chronic mucosal inflammation with cryptitis and crypt abscesses, accompanied by prominent eosinophilic infiltration. No granulomatous changes or vasculitis were observed.

**Figure 2:**
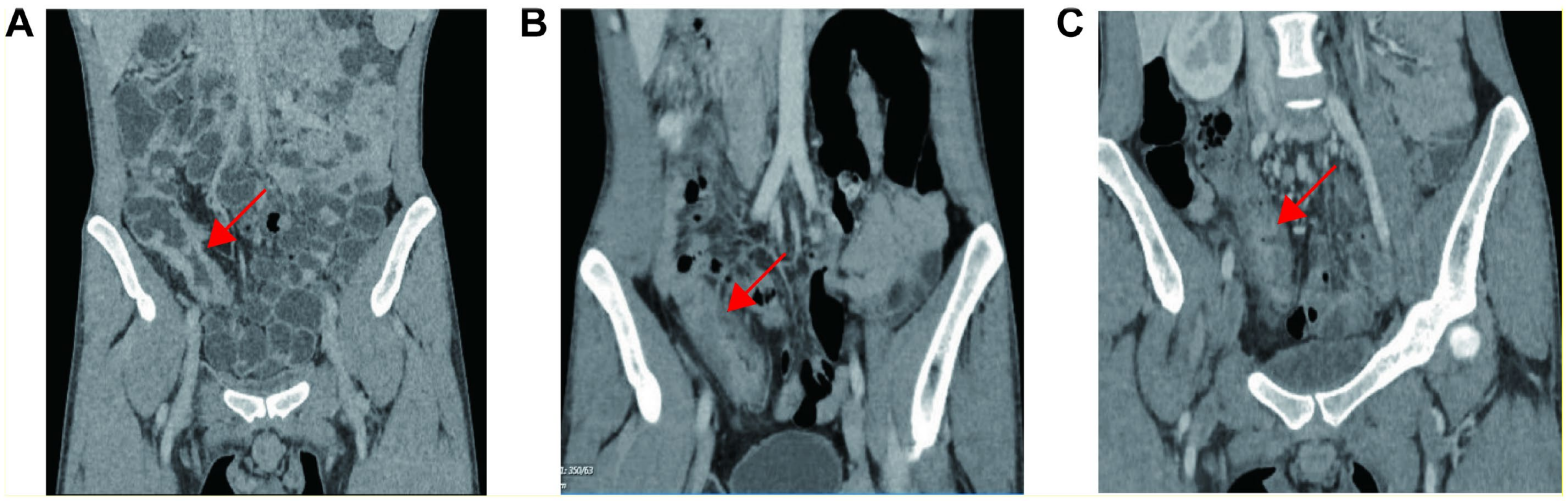
Serial radiologic assessment of ileocecal involvement. **(A)** Contrast-enhanced CT at baseline demonstrated marked bowel wall thickening and luminal narrowing (arrows). **(B)** After infliximab induction (week 14) residual stenosis persisted (arrows). **(C)** Following therapeutic drug monitoring–guided infliximab dose intensification (every 4 weeks × 2), no further radiologic improvement was evident.

Baseline CT demonstrated segmental bowel wall thickening and luminal stenosis involving the terminal ileum and cecum, with associated appendiceal wall thickening and multiple enlarged mesenteric lymph nodes (Figure 2A). Repeat pathology review and special staining excluded granulomas, vasculitis, or specific infectious pathogens. Immunohistochemistry results were: CMV (−), EBER (−), CD3 (+), CD20 (+), Ki-67 positive in ~20% of lymphocytes, KP-1 (+), and CD56 (−). PAS, acid-fast, silver, and elastic fiber staining were all negative.

A multidisciplinary consultation involving gastroenterology, rheumatology, ophthalmology, and oral medicine supported the diagnosis of systemic Behçet’s disease (BD), with involvement of the oral cavity, genital region, skin, and gastrointestinal tract. The diagnostic reasoning integrated systemic mucocutaneous features (recurrent oral and genital ulcers with acneiform lesions) and ileocecal stricturing ulcerative disease, while systematically excluding mimics. Infectious causes were considered but were not supported by negative microbiologic testing and special stains. Crohn’s disease remained in the differential given ileocecal involvement and perianal disease history; however, serial biopsies showed no granulomas, and the overall clinical synthesis favored intestinal BD.

The patient was started on infliximab (IFX) at a dose of 5 mg/kg intravenously, administered at weeks 0, 2, 6, and 14. After three doses, oral ulcers significantly improved, and abdominal pain was alleviated. Repeat CT enterography performed after infliximab induction (week 14) showed residual ileocecal luminal stenosis persisted (Figure 2B). However, by week 10, the patient developed transient fever, abdominal pain, and diarrhea. At week 14, therapeutic drug monitoring (TDM) revealed a low IFX trough concentration (1.5 μg/mL; reference range: 3–7), no detectable anti-drug antibodies, and a markedly elevated TNF-*α* level (47.25 pg/mL; normal <10).

The treatment regimen was intensified to IFX every 4 weeks. After two additional doses, the drug concentration rose to 16.4 μg/mL and TNF-α decreased to 10.09 pg/mL, with transient clinical improvement. Anti-drug antibodies remained undetectable on repeat testing after intensification. Despite this transient symptomatic improvement, repeat imaging did not demonstrate further radiologic resolution of the ileocecal stenosis after therapeutic drug monitoring–guided infliximab dose intensification (Figure 2C). However, before the sixth infusion, the patient again experienced abdominal pain, diarrhea, oral ulcers, and intermittent fever, suggesting secondary loss of response to infliximab despite TDM-guided dose intensification.

Following re-evaluation, treatment was switched to oral upadacitinib at 45 mg daily. The patient’s symptoms improved rapidly after initiation: abdominal pain and diarrhea resolved, oral ulcers disappeared, and no further febrile episodes occurred. Within approximately 2 weeks, he reported significant improvement in appetite and energy. Corticosteroids were discontinued without symptom rebound.

**Figure 3:**
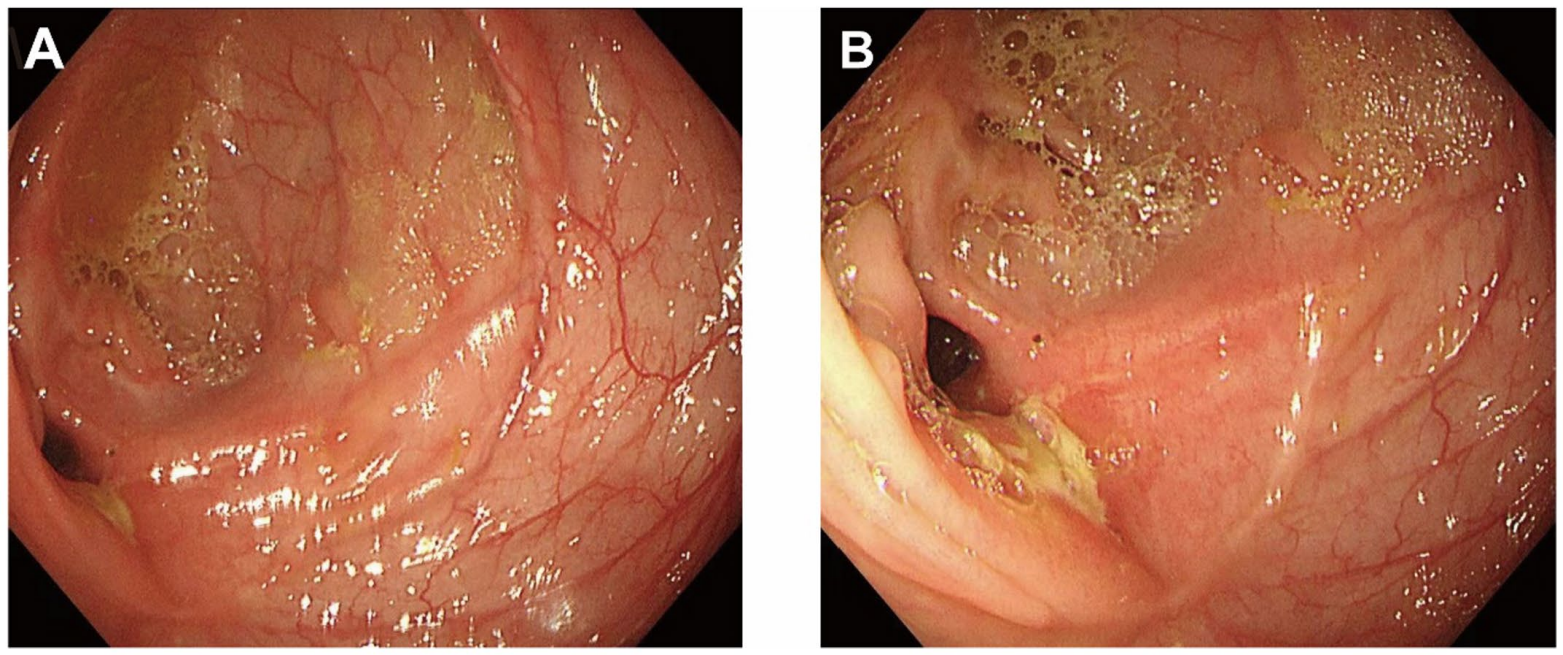
Follow-up colonoscopy showing mucosal healing at the previously ulcerated site **(A, B)**.

During follow-up, regular monitoring of blood counts, inflammatory markers, and TNF-*α* levels demonstrated continuous improvement. Hemoglobin normalized, and body weight increased from 52 to 75 kg. BMI returned to the normal range. Follow-up colonoscopy showed complete mucosal healing at the lesion site, with no residual stricture or new ulceration (Figure 3).

The patient continued maintenance therapy with upadacitinib and remained in sustained clinical remission for over 6 months. No adverse events were reported, and his quality of life markedly improved.

## Discussion

Behçet’s disease is a relapsing, chronic vasculitis that affects multiple organ systems and presents with considerable variability in clinical expression. Among its diverse manifestations, intestinal involvement is one of the most severe, often producing gastrointestinal symptoms that closely mimic Crohn’s disease, thereby complicating accurate diagnosis and therapeutic decision-making (8). The patient in this report—a young adult male—exhibited persistent postprandial abdominal pain, progressive weight loss, and hallmark mucocutaneous signs such as oral and genital ulcerations and perianal abscesses. Colonoscopic evaluation revealed extensive circumferential ulceration with luminal narrowing. Histological analysis demonstrated cryptitis, crypt abscesses, and marked eosinophilic infiltration without granulomatous changes or vasculitis, favoring a diagnosis of BD over CD. Multidisciplinary consensus supported systemic BD with gastrointestinal tract involvement (9).

The absence of pathognomonic histologic findings and specific serological markers renders the diagnosis of intestinal BD challenging. It necessitates diagnosis through clinical synthesis and the exclusion of mimicking conditions like infectious colitis, intestinal tuberculosis, and idiopathic inflammatory bowel disease. While vasculitis is a classical histopathologic feature of BD, it is seldom identified in gastrointestinal biopsy specimens due to the superficial location of ulcerative lesions. In this case, serial biopsies and targeted staining techniques excluded infectious and granulomatous causes, further reinforcing the diagnosis of BD (10).

Initial management of intestinal BD usually consists of corticosteroids in combination with immunomodulatory agents such as thalidomide or azathioprine. In cases with moderate to severe or treatment-resistant disease, biologics—particularly TNF-*α* antagonists—have been adopted with increasing success. Both infliximab (IFX) and adalimumab (ADA) have been shown to induce clinical remission and support mucosal healing (11). According to pooled data from meta-analyses, remission rates can reach up to 50% within 3 months, and mucosal healing exceeds 60% at 12 months. In prospective studies, IFX efficacy in intestinal BD has been evaluated using predefined timepoints and structured criteria combining symptoms with objective examinations. In the multicenter phase 3 study by Hibi et al. (12), clinical response was explored as early as week 14 and the primary efficacy assessment was performed at week 30; dose escalation was considered after week 30 in selected patients with inadequate response or loss of response, supported by symptom-based tools such as a visual analogue scale. In BIO-BEHÇET’S, overall Behçet’s disease activity was quantified using the Behçet’s Disease Activity Index (BDAI) at weeks 12 and 24 (13). Although validated indices such as the Disease Activity Index for Intestinal Behçet’s Disease (DAIBD) are available, DAIBD/BDAI/VAS scores were not prospectively recorded at each visit in this case; treatment escalation and eventual transition were guided by recurrent clinical symptoms together with objective inflammatory markers and proactive therapeutic drug monitoring (trough infliximab level and anti-drug antibodies), and were corroborated by subsequent endoscopic reassessment. However, this patient did not respond adequately to standard-dose IFX (14). Therapeutic drug monitoring revealed low trough concentrations of IFX and elevated TNF-*α*, indicative of insufficient pharmacodynamic effect rather than immunogenic failure. Despite dose intensification, clinical relapse occurred, consistent with secondary loss of response and insufficient pharmacodynamic effect rather than immunogenic failure (15).

Due to this inadequate therapeutic effect, the patient was switched to upadacitinib (UPA), a selective Janus kinase 1 (JAK1) inhibitor. UPA (ABT-494) is designed to preferentially target JAK1 with the goal of optimizing therapeutic outcomes and minimizing off-target side effects. Although not yet an established treatment for BD, small-scale reports and limited studies suggest its potential benefit. In this case, UPA achieved rapid and durable symptom control, normalization of inflammatory biomarkers, significant weight recovery, and endoscopic evidence of mucosal healing. Corticosteroids were successfully withdrawn without clinical deterioration, and no adverse effects were recorded during follow-up (16).

While the patient achieved mucosal remission following upadacitinib therapy, it is important to acknowledge a key limitation: a colonoscopic assessment was not performed immediately prior to initiating this treatment. Therefore, a definitive causal attribution to JAK inhibition alone cannot be made. However, given the patient’s persistent clinical symptoms, elevated inflammatory markers, and radiologic evidence of active disease following anti-TNF failure and dose intensification (with documented supratherapeutic drug levels), it is highly unlikely that spontaneous mucosal remission had occurred at that juncture. This inference, while reasonable, remains unconfirmed and constitutes a notable limitation of our case report. Further longitudinal research and real-world clinical data are essential to define the role of agents like upadacitinib within the treatment paradigm for intestinal BD (16, 17).

## Conclusion

This case highlights the diagnostic and therapeutic challenges associated with intestinal Behçet’s disease in the era of biologics. The patient exhibited typical gastrointestinal and mucocutaneous features of BD but failed to respond to both standard and intensified doses of infliximab. Therapeutic drug monitoring (TDM) supported loss of response to infliximab, prompting a switch to the selective JAK1 inhibitor upadacitinib. The patient experienced rapid symptom relief, complete mucosal healing on endoscopy, and sustained steroid-free remission for over 6 months without any adverse events. This case adds to emerging evidence that selective JAK1 inhibition may be an effective option for anti-TNF–refractory intestinal Behçet’s disease. Larger studies with longer follow-up are needed to define long-term efficacy and safety.

## Data Availability

The original contributions presented in the study are included in the article/supplementary material, further inquiries can be directed to the corresponding author.

## References

[1] Emmi G Bettiol A Hatemi G Prisco D. Behçet's syndrome. Lancet. (2024) 403:1093–108. doi: 10.1016/S0140-6736(23)02629-6, 38402885

[2] Belfeki N Ghriss N Le Joncour A Saadoun D. Etiopathogenesis of Behçet's disease: a systematic literature review. Clin Immunol. (2025) 279:110549. doi: 10.1016/j.clim.2025.110549, 40571238

[3] Liu S Pan Q Xu H Luo Y. Activation of fibroblast in intestinal Behçet's disease shown by 68Ga-FAPI-04 PET/CT. Nuklearmedizin. (2025) 64:231–3. doi: 10.1055/a-2543-0982, 40418934

[4] Zheng Y Kong G Guo H Liu Z Yan K. Multiple aseptic abscesses and pulmonary involvement in a child with Behcet's disease phenotype: a case report. Front Immunol. (2025) 16:1550551. doi: 10.3389/fimmu.2025.1550551, 40552301 PMC12183221

[5] Xu W Wu X Yu Q Sun K Mao X Ye H . Predictors of anti-TNF treatment failure in intestinal Behcet's disease: a multicenter retrospective cohort study. Clin Rheumatol. (2025) 44:3655–65. doi: 10.1007/s10067-025-07581-y, 40668289

[6] Park D Park J Park SJ Park JJ Kim TI Cheon JH. Impact of body mass index on clinical outcomes in intestinal Behcet's disease. Korean J Intern Med. (2025) 40:606–15. doi: 10.3904/kjim.2024.349 40635487 PMC12257004

[7] Gao T Ren X Han Y Wang F Liu B Fang Y. Therapeutic effect of adalimumab in the treatment of intestinal Behcet's disease in children. Front Pediatr. (2025) 13:1619065. doi: 10.3389/fped.2025.1619065 40661890 PMC12256472

[8] Tian H Ye J Li Z Zhao J. Appendix vasculitis in a patient with intestinal Behçet's disease. Arthritis Rheumatol. (2026) 78:257–58. doi: 10.1002/art.43331 40665781

[9] Giorgio CM Di Brizzi EV Licata G Fiorentino C Argenziano G Damiani L. Dermatological insight as the key to diagnosing intestinal Behçet's disease misdiagnosed as Crohn's: a case report. Dermatol Rep. (2025) 17:10232. doi: 10.4081/dr.2025.10232 PMC12448051 39901835

[10] Hou CC Bao HF She CH Chen HY Pan GX Chen HN . Specific plasma metabolite profile in intestinal Behçet's syndrome. Orphanet J Rare Dis. (2025) 20:21. doi: 10.1186/s13023-024-03484-4, 39806438 PMC11727545

[11] Li Wai Suen CFD Choy MC Con D Cheng K Nigro J Breheney K . Early infliximab levels and clearance predict outcomes after infliximab rescue in acute severe ulcerative colitis: results from PREDICT-UC. Gastroenterology. (2026) 170:118–31. doi: 10.1053/j.gastro.2025.07.020 40701396

[12] Hibi T Hirohata S Kikuchi H Tateishi U Sato N Ozaki K . Infliximab therapy for intestinal, neurological, and vascular involvement in Behcet disease: efficacy, safety, and pharmacokinetics in a multicenter, prospective, open-label, single-arm phase 3 study. Medicine (Baltimore). (2016) 95:e3863. doi: 10.1097/MD.0000000000003863, 27310969 PMC4998455

[13] Moots RJ Fortune F Jackson R Thornburn T Morgan A Carr DF . Infliximab vs interferon-α in the treatment of Behçet's syndrome: clinical data from the BIO-BEHÇET'S randomized controlled trial. Rheumatology (Oxford). (2025) 64:2882–91. doi: 10.1093/rheumatology/keae585, 39432564

[14] Harte M Macken J Zou L Fortune F. Case report: vedolizumab in oral Crohn's disease: the downsides of a gut-specific therapy for a multi-site disease. Front Med. (2024) 11:1485394. doi: 10.3389/fmed.2024.1485394, 39697207 PMC11653175

[15] Kim SJ Park EJ Bae HW Lee YJ Park MY Yang SY . Risk factors of reoperation in patients with intestinal Behçet's disease treated by initial bowel resection. J Clin Med. (2024) 13:6771. doi: 10.3390/jcm13226771, 39597915 PMC11594750

[16] Sha S Xu B Wang K Qiao C Shi H Jiang J . Case report: successful remission with upadacitinib in a young patient with anti-TNF-refractory intestinal Behçet's disease. Front Immunol. (2024) 15:1483993. doi: 10.3389/fimmu.2024.1483993, 39582862 PMC11582029

[17] Kraev K Uchikov P Hristov B Kraeva M Basheva-Kraeva Y Popova-Belova S . Coexistence of ankylosing spondylitis and Behçet's disease: successful treatment with upadacitinib. Immun Inflamm Dis. (2024) 12:e1242. doi: 10.1002/iid3.1242, 38578007 PMC10996370

